# Analysing Head-Thorax Choreography During Free-Flights in Bumblebees

**DOI:** 10.3389/fnbeh.2020.610029

**Published:** 2021-01-12

**Authors:** Luise Odenthal, Charlotte Doussot, Stefan Meyer, Olivier J. N. Bertrand

**Affiliations:** ^1^Neurobiology, University Bielefeld, Bielefeld, Germany; ^2^Department of Informatics, University of Sussex, Brighton, United Kingdom

**Keywords:** bees, machine learning, random forest, decision tree, neural network, coordination, control, active vision

## Abstract

Animals coordinate their various body parts, sometimes in elaborate manners to swim, walk, climb, fly, and navigate their environment. The coordination of body parts is essential to behaviors such as, chasing, escaping, landing, and the extraction of relevant information. For example, by shaping the movement of the head and body in an active and controlled manner, flying insects structure their flights to facilitate the acquisition of distance information. They condense their turns into a short period of time (the saccade) interspaced by a relatively long translation (the intersaccade). However, due to technological limitations, the precise coordination of the head and thorax during insects' free-flight remains unclear. Here, we propose methods to analyse the orientation of the head and thorax of bumblebees *Bombus terrestris*, to segregate the trajectories of flying insects into saccades and intersaccades by using supervised machine learning (ML) techniques, and finally to analyse the coordination between head and thorax by using artificial neural networks (ANN). The segregation of flights into saccades and intersaccades by ML, based on the thorax angular velocities, decreased the misclassification by 12% compared to classically used methods. Our results demonstrate how machine learning techniques can be used to improve the analyses of insect flight structures and to learn about the complexity of head-body coordination. We anticipate our assay to be a starting point for more sophisticated experiments and analysis on freely flying insects. For example, the coordination of head and body movements during collision avoidance, chasing behavior, or negotiation of gaps could be investigated by monitoring the head and thorax orientation of freely flying insects within and across behavioral tasks, and in different species.

## 1. Introduction

Animals travel in their habitat to chase prey, escape predators, find mates, or food. The motile body parts, such as legs, wings, or fins, often differ from the sensory ones. For example, the eyes of most sighted animals are placed on the head, away from wings or legs. The non-collocation of motile and sensory body parts allows many animal species to decouple where to look and where to move. Notably, animals frequently stabilize their head while traveling in their environment to compensate for body motion (e.g., roll movements) that is required for steering (e.g., Van Hateren and Schilstra, 1999a; Ravi et al., 2016) or actively move their head to extract relevant information, for example the distance to a prey or landing site (Sobel, 1990; Kral, 2003, 2012). Adequate motion of an animal in its habitat and perception of its surrounding requires the well-coordinated orchestration of sensory and motile body parts.

Flying insects orchestrate their flight similarly to ballet dancers performing a chainé or a pirouette. They first start turning their thorax at a slow speed, and then later turn their head at a higher speed. Between such sharp head turns, the head direction is mostly stabilized (Van Hateren and Schilstra, 1999a,b; Viollet and Zeil, 2013; Doussot et al., 2020b; Verbe et al., 2020) allowing flying insects to estimate the distance to neighboring objects (Srinivasan, 2011; Kern et al., 2012), traveled distance (Srinivasan, 2011), perceive gaps (Ravi et al., 2019), or land (Frasnelli et al., 2018) by using the apparent motion of nearby objects on their retina (Egelhaaf et al., 2014). This active gaze strategy requires excellent coordination between the head and thorax, respectively. However, due to the small size of flying insects, head-body coordination has been analyzed rarely and most studies have focused on the insect's thorax orientation.

The thorax orientation of insects gives only a poor proxy of the viewing direction (Van Hateren and Schilstra, 1999a,b; Riabinina et al., 2014; Doussot et al., 2020b). Therefore, recordings lacking head orientation information limit our understanding of the perception-behavior loop. However, in flying insects, one crucial aspect of their perception takes place between sharp head turns, i.e., during intersaccades (Egelhaaf et al., 2014). Therefore, by predicting the occurrence of the head's saccades from the time course of thorax orientation, we could deepen our understanding of the behavior of flying insects.

We used recordings of the head and thorax orientation of free-flying bumblebees *Bombus terrestis* (Doussot et al., 2020a), that include footage of high spatial and temporal resolution and from different perspectives to develop methods to lessen such limitations.

In most previous experiments, only the orientation of the thorax could be determined, due to technological limitations. We developed a method to locate the head intersaccades solely from the time course of thorax orientation. We based our method on classifiers (decision tree and random forest) and tested our approach in two scenarios often encountered in experimental design. First, many insect flights are recorded at frame rates lower than 500 fps. Second, many recordings only report the orientation along one axis of rotation (often the z-axis) (for example, Kern et al., 2012; Lobecke et al., 2018; Robert et al., 2018; Lecoeur et al., 2019; Ravi et al., 2019). However, the orientation of an animal is defined around three axes. Thus, we tested our classifier with only the orientation around the z-axis.

Our first method focused on the saccade/intersaccade classification. We developed a second method to predict the detailed time course of head and thorax orientation, elaborating on an approach developed by Dürr and Schilling (2018) using an artificial neural network to map the posture of one leg of the stick insects to another. We extended their method by adding a temporal component (forecasting or backcasting) and applied it to our bumblebees' flight. Our approach may serve as a computational ground plan for investigating body part coordination in other animals.

## 2. Materials and Methods

### 2.1. Data Acquisition

#### 2.1.1. Animal Preparation

**Figure 1:**
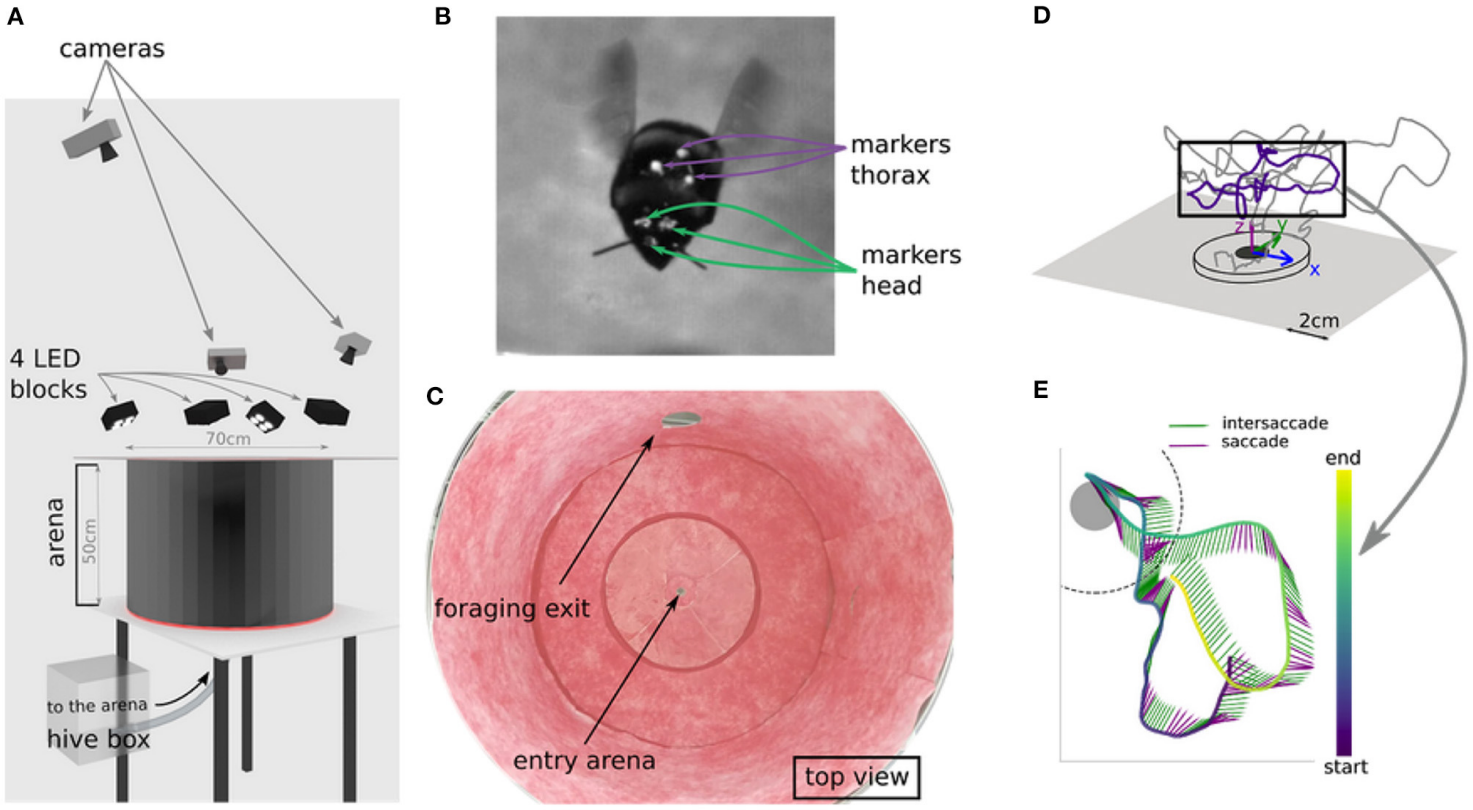
Experimental setup and example flight. **(A)** Virtual setup of the arena. Bee enters the flight arena through the hole at the bottom, that connects to the hive. **(B)** Marker positions on head and thorax. **(C)** Top view of real world arena. **(D)** Example of a learning flight in 3d space. **(E)** Temporal structure of a section of a leaning flight as lollipop plot, with each sticks pointing direction indicating thorax orientation and color indicating movement classification (green = intersaccade; purple = saccade).

Data were collected according to Doussot et al. (2020a). We explain the procedure here for clarity. We used a healthy hive of *Bombus terrestris* provided by Koppert B.V., The Netherlands. Bumblebees were manually marked and transferred into a 30 × 30 × 30 cm acrylic box. Marking the head was done by painting three small dots (~ 1 mm diameter each) with acrylic paint on the bees' heads: one above each eye and the one in between the eyes at the height of the antenna scape insertion point. Special attention was paid to not cover the ocelli and the eyes of the bumblebees (Figure 1B). We marked the thorax by fixing an equilateral triangle (side length of 5 mm) of black paper with a white pearl dot (1 mm diameter) at each apex with wax.

The bumblebees entered a cylindrical flight arena with a radius of 35 cm and a height of 50 cm through a 1 cm hole in the center of the ground (Figures 1A,C). The flight arena was connected to a foraging chamber.

#### 2.1.2. Tracking of Head and Thorax Markers

Six learning flights of bees were recorded using three synchronized high-speed cameras (Optronis CR3000x2) with a resolution of 1,710 × 1,696 pixels. The three cameras sampled a volume of ~ 10 × 10 × 10 cm^3^ around the nest entrance from different perspectives. The recording area was restricted to a small part of the arena as we intended to monitor the head and thorax orientation at a high spatial resolution. The recorded volume was illuminated by four blocks of four LEDs each (HIB Multihead LED, HS vision GmbH, Germany).

We started the recordings as soon as a marked bumblebee took off. Recordings were made at a shutter speed of 1/2, 000 s, a frame rate of 500 frames per second, and for ~ 11 s. The three cameras were calibrated using the Matlab toolbox dltdv5 (Hedrick, 2008).

We developed and assessed our method based on six learning flights of marked bees. Tracking of head and thorax markers was achieved with a custom-made Python script, based on OpenCV. The videos were then manually reviewed with the software IVtrace (https://opensource.cit-ec.de/projects/ivtools) to ensure correct detection. We then reconstructed the marker positions in 3D space using the Matlab toolbox dltdv5 (Figures 1D,E).

#### 2.1.3. Orientation

Given a three dimensional space, one can infer the orientation of any solid object in it, by defining an object-specific coordinate system of three orthogonal unit vectors centered at a pivot point. One then can describe the orientation of this object by the relative orientation of this object-specific coordinate system with respect to the world coordinate system. In order to describe the orientation of the bees' head and thorax at any given time, we first chose an appropriate coordinate system for our arena and then reconstructed the bee coordinate system from the markers we placed on them.

The head (resp. thorax) coordinate system was defined as follows. Its origin was defined as the center of mass of the three markers placed on the head (resp. thorax). Two of the three markers were aligned with from left to right in the head (resp. thorax) coordinate system, and thus formed the y-vector. The x-vector was orthogonal to the y-axis and passed by the third marker. Finally, the z-vector being orthogonal to the two other axes were computed as cross product between the x and y-vector. In mechanics, the orientation of a solid object is often defined by three rotational angles (Euler angles). Different conventions can be used to define the rotational angles. The conventions differ by the order of elementary rotations. Here, we used the yaw-pitch-roll convention (Diebel, 2006). This convention is defined as rotating first around the roll axis (x-axis in the world coordinate system), then around the pitch axis (y-axis of a temporary coordinate system), and finally around the yaw axis (z-axis in the head or thorax coordinate system). This transformation was performed at each instant of time, yielding the time courses of the yaw, pitch, and roll angles for the head and the thorax.

### 2.2. Saccade and Intersaccade Classification

#### 2.2.1. Ground Truth: Thresholding on Head's Orientation

The YPR orientation was filtered with a one-dimensional-cubic spline function (with smoothing parameter λ = 150) (Scipy.signal). The smoothing parameter λ, interpreted as the degree of freedoms, was estimated from a generalized cross-validation criterion with R (see Supplementary Figure 1 for the effect of lambda on our method). Cubic splines are often used in biomechanics data filtering (Woltring, 1985), since abrupt changes in the data are not smoothed out, in contrast to low pass filtering. Based on the angular velocity of the head around the z-axis ω_*z*_(*t*) in the bee coordinate system, intersacades and saccades were extracted using a two-thresholds method. For derivatives higher than 372.42°/*s* (manually determined), the time point was considered as being part of a saccade. The neighboring time points were considered part of the same saccade, if the derivative was higher than 200.54°/*s*, and as part of an intersaccade otherwise.

#### 2.2.2. Benchmark: Thresholding on Thorax's Orientation

In numerous experiments, the orientation of the bee's head cannot be resolved. Thus, researchers usually segregate the trajectories into saccades and intersaccades by using a threshold on the thorax angular velocities. The threshold is usually chosen by observing the variation of the thorax angular velocity over time (Van Hateren and Schilstra, 1999a; Riabinina et al., 2014; Mertes et al., 2015). Here, head orientation data was available. Thus, instead of choosing the threshold by visually observing the time course of the thorax's angular velocity, we chose them such that the accuracy of the classification is maximized i.e., the number of true positives (a time point *t*, head and thorax are saccade), and true negative (a time point *t*, head and thorax are intersaccade). The thorax's saccades extracted by this method are the benchmark for our classifier method.

#### 2.2.3. Classifier Based Method

We investigated whether non-single-threshold classifiers can outperform the segregation based on hard thresholding of the thorax's angular velocity (i.e., our benchmark). Hence we compared the established benchmark (Th) with two well-known non-linear classifiers: a decision tree (DT) and a random forest (RF). A decision tree can have a high variance in the optimal tree. The use of a random forest, i.e., multiple trees, reduces the variance in the optimal classifier. We define the learning task as follows: Given a finite time series of angular z-velocities obtained from the thorax, predict the binary class label associated with the center measurement of this time series, where labels are “head saccade” or “head intersaccade.”

**Figure 2:**
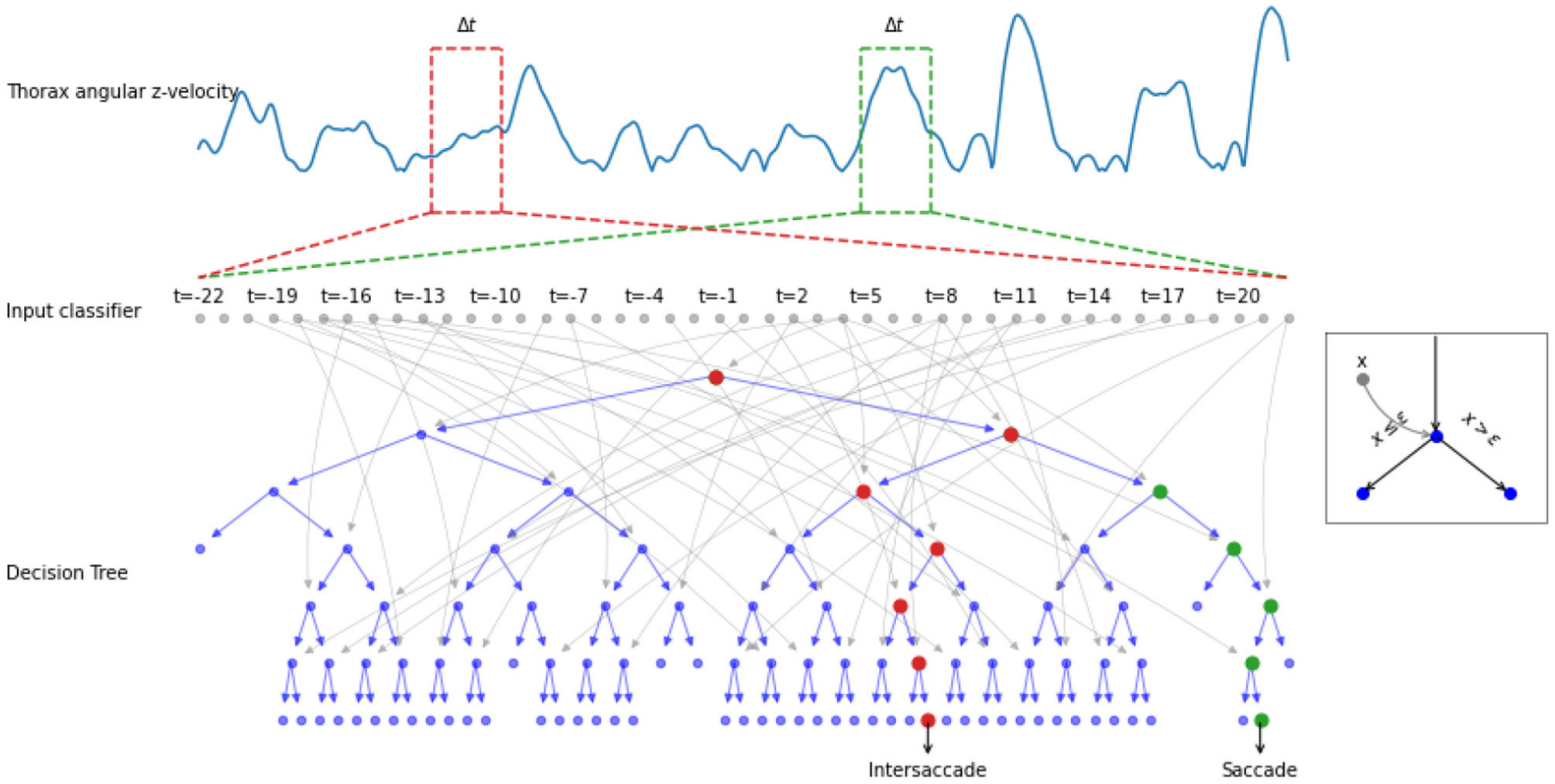
Example of the decision process done by a decision tree. The thorax angular z-velocity (in blue) within a given time window Δ*t* (e.g., red and green dotted rectangle), is the input of the classifier. A decision tree is composed of nodes (circles), with input *x*_*i*_ (gray arrows). If the input *x*_*i*_ is greater or equal to a learned threshold ϵ, the right node is selected, otherwise the left (see example in the gray square). The process is repeated until a decision saccade/intersaccade can be drawn. The red (resp. green) node highlights the decision path for an intersaccade (resp. for a saccade).

More formally, the input of the classifier was thus $\Omega_{z}^{\text{thorax}}\left(t\right)=\left[\omega_{z}\left(t-\Delta t/2\right),\cdots \;,\omega_{z}\left(t+\Delta t/2\right)\right]$ with Δ*t* being the time window (a hyperparameter of our classifier). Hence, the classifier yields a class *C*(*t*) ∈ ℂ = 0, 1 by applying a function *f* :ℝ^⋉^ → ℂ on the input $\Omega_{z}^{\text{thorax}}\left(t\right)$. We have, thus, the equation: $C\left(t\right)=f\left(\Omega_{z}^{\text{thorax}}\left(t\right)\right)$ (Figure 2). *C*(*t*) = 0 [resp. *C*(*t*) = 1] means that the angular velocity at the timepoint belongs to an intersaccade (resp. saccade).

The classifiers were trained, validated, and tested using scikit-learn (Pedregosa et al., 2011). The training was used to adjust the parameters of the classifier. The training set consisted of the first 70% of samples per learning flight for all 5 learning flights. The validation was used to select hyperparameters: depth *D* (i.e., the number of layers in a Decision Tree) and the time window Δ*t*, by varying the parameters systematically *D* ∈ [1, 20], and Δ*t* ∈ [0, 50] ms. The validation set consisted of the remaining 30% of the five flights used for the training set. Since our classes are sufficiently balanced, we used the accuracy of the classifier to determine which classifier performed best on the validation/test data set.

To test the performance of the best classifiers, we used one learning flight that was neither used for training nor for the test (i.e., forming the validation data set). The trained saccade-intersaccade classifier was applied for every time point. The prediction *C*(*t*), saccade, or intersaccade, at time *t* was compared to the ground truth *H*(*t*) obtained by thresholding the head's angular velocity. Our goal was to outperform the classification from a benchmark, namely the classification *T*(*t*) based on the thresholding of the thorax's angular velocity. We thus compared the accuracy of the classifier ACC_classifier_ with the accuracy of the benchmark ACC_benchmark_. ACC_classifier_ and ACC_benchmark_ are defined by:

(1) $$\begin{array}{r} \;{\text{ACC}}_{\text{classifier}}=\frac{\sum_{t}\left[C\left(t\right)==H\left(t\right)\right]}{N}\\ {\text{ACC}}_{\text{benchmark}}=\frac{\sum_{t}\left[T\left(t\right)==H\left(t\right)\right]}{N} \end{array}$$

Here [⋯ ] are the Iverson brackets. The Iverson brackets is a notation that takes a true/false input. Let *P* be a true/false statement. [*P*] is defined to be 1 if *P* is true, and 0 otherwise. *N* is the number of time points in the learning flights.

#### 2.2.4. Extension: Often Encountered Situations

To further assess the validity of our method, we investigated the accuracy of our classifier in two often encountered situations. First, researchers are not always able to record at a high frame rate. Second, some behavioral assays rely on single-perspective recordings, and therefore the orientation of the thorax cannot be determined entirely. Assumptions need to be made about certain axis of rotations. For example, when the orientation of the body long-axis is derived from a top view camera, it is often assumed that the orientations pitch and roll are null. To investigate the robustness of the classifier at a lower-frame rate, we down-sampled our recordings and interpolated them by using a cubic spline in order to recover the 500 fps on which the classifiers are trained. The classification was then performed on the angular velocities of the thorax derived from downsampled and interpolated trajectories. To investigate the impact of a null-pitch and null-roll assumption, we set the z positions of the markers at a given frame to their average z position (mimicking top view recordings). The orientation was then calculated yielding only the variation of yaw. The accuracy of the classifiers was then calculated on the angular velocities (here equivalent to the derivative of the yaw orientation, because pitch and roll are null).

### 2.3. Predicting Head Angular Velocity From Thorax

The time course of the head and thorax position are tightly linked. However, the head can be in a different orientation as the body thanks to the neck muscles connecting the two. In flying insects, the time course of the head angular velocity appears to be loosely correlated with the thorax angular velocity. Indeed when the head is rotating fast, the thorax is likely to turn quickly as well (Kern, 2006). In other words, the head and thorax angular velocities share some information that may be used to predict the one from the other (e.g., predict the head angular velocity from the thorax angular velocity).

Predicting head angular velocity from thorax angular velocity in a reliable manner could allow researchers to record only the thorax orientation to study the head orientation, alleviating the need to mark the head and monitoring it with high-resolution cameras. Our method focuses on predicting the angular velocity around the z-axis, because the angular velocity is varying the most around this axis during bee learning flights. The prediction of the head angular velocity along the z-axis will be based on the body angular velocity during a time interval Δ*t*:

(2) $$\begin{array}{r} {\hat{\omega }}_{z}^{\text{head}}\left(t\right)=g\left(\omega_{z}^{thorax}\left(t-\Delta t/2\right),\cdots \;,\omega_{z}^{thorax}\left(t-\Delta t/2\right)\right) \end{array}$$

Where $\omega_{z}^{\text{thorax}}\left(t\right)$ is the instantaneous angular velocity of the thorax around the z-axis at time *t*. ${\hat{\omega }}_{z}^{\text{head}}\left(t\right)$ is the prediction of the instantaneous angular velocity of the head around the z-axis at time *t*. *g*():ℝ^*n*^ → ℝ is a function (e.g., a neural network) used for prediction.

Predicting the motion of one body part from another (e.g., head angular velocity from thorax angular velocity), could inform about the predictive causality between the two body-parts and therefore the underlying control mechanisms (Granger, 1969). Thus, we predicted the thorax angular velocity along the z-axis based on the head angular velocity during a time interval Δ*t*. Our method will, therefore, be described for predicting the head angular velocity from thorax angular velocity.

#### 2.3.1. Neural Network Architecture

To predict the motion of one body part from another, we used a feed-forward artificial neural network. The neural network consisted of three layers. The input layer contained as many neurons as measures of instantaneous angular velocity within the time window Δ*t* plus a bias neuron (acting in a similar manner as the intercept in a linear fit). So for recording at 500 fps and Δ*t* express in *ms*: 1+0.5Δ*t* neurons. The second layer, i.e., the hidden layer, contains *N*+1 neurons with *N* ∈ 1, 2, 4, 8, 16, 32, 64, 128. The activation functions of the units were rectified linear (relu). A neuron with a relu activation function will have an output proportional to its input when the input is positive. However, when the input is negative, the neuron will output zero. The last and third layer contained two output neurons with a hyperbolic tangent activation function. A neuron with an hyperbolic tangent activation with an input *x* will output tanh*x*. The two neurons encoded the sine and cosine of the predicted angular velocity around the z-axis, and their response at time *t* will be referred to as *O*_*s*_(*t*) and *O*_*c*_(*t*), respectively.

The neural network has two hyperparameters: the number of neurons in the hidden layers *N* and the size of the time window Δ*t*. To find the optimal *N* and Δ*t*, we performed a grid search over the parameter space with Δ*t* ∈ {1, 3, 5, ⋯ , 53}ms and *N* ∈ {1, 2, 4, 8, 16, 32, 64, 128} resulting in 40 neural networks.

#### 2.3.2. Training

The neural networks were implemented and trained using tensorflow API for python (Abadi et al., 2015). The weights of the networks were randomly initialized. To train the network we used the Adam optimizers (Kingma and Ba, 2015) with the loss function that the training procedure aim at minimizing:

(3) $$\begin{array}{r} L=\underset{t}{\sum }\frac{|\left(\begin{array}{c} \cos\left(\omega_{z}^{\text{head}}\left(t\right)\right)\\ \sin\left(\omega_{z}^{\text{head}}\left(t\right)\right) \end{array}\right)-\left(\begin{array}{c} O_{c}\left(t\right)\\ O_{s}\left(t\right) \end{array}\right){|}_{2}}{\lambda |\left(\begin{array}{c} \cos\left(\omega_{z}^{\text{thorax}}\left(t\right)\right)\\ \sin\left(\omega_{z}^{\text{thorax}}\left(t\right)\right) \end{array}\right)-\left(\begin{array}{c} O_{c}\left(t\right)\\ O_{s}\left(t\right) \end{array}\right){|}_{2}+\epsilon } \end{array}$$

The numerator in the loss contains the euclidian norm of a vectorial difference. The vector ${\left(\cos\left(\omega_{z}^{\text{head}}\left(t\right)\right),\sin\left(\omega_{z}^{\text{head}}\left(t\right)\right)\right)}^{T}$ is the direction of the bee's head velocity expressed in Cartesian coordinates. The vector ${\left(O_{c}\left(t\right),O_{s}\left(t\right)\right)}^{T}$ is formed by the two output neurons of our network. The euclidian norm of the vectorial difference can therefore provide of a measure of the network performance, because when the two vector match their difference is a null vector. In the loss function, the numerator (resp. denominator) decreases as the output of the network approaches the angular velocity of the head (resp. thorax). The loss function is thus small when the prediction is close to the head angular velocity and far from the thorax angular velocity. The denominator, thus, guarantees that when the prediction is close to the thorax angular velocity, the loss function is high, decreasing the risk of the network learning the identity, i.e., predicting the thorax from the thorax. λ is a regularization term and is equal to 0.5. ϵ is a small value to avoid division by zero and is equal to 0.1. The networks were trained for 30 epochs on the first 70% of each of the five learning flights.

#### 2.3.3. Choosing Hyperparameters and Validation

To choose the hyperparameters Δ*t* and *N* of our predictive method, we evaluated the performance of the network on the remaining 30% of the five learning flights (i.e., on the test data set). From the 40 trained networks per hyperparameter tuple, we calculated the unsigned error angle ΔΩ between predicted head angular velocity ${\hat{\omega }}_{z}^{H}\left(t\right)$ and the measured head angular velocity $\omega_{z}^{H}\left(t\right)$ over time. The hyperparameters yielding the smallest median unsigned error angle were retained for validation (Supplementary Figure 2 and Supplementary Table 1).

To assess the performance of our predictive method, we use the previously trained neural networks on data never seen by the networks. We use the sixth recorded learning flight. The thorax to head prediction the optimal number of neurons is 32 and the optimal window size is 29. For the head to thorax prediction we have an optimal number of neurons of 4 and the optimal window size is 45.

#### 2.3.4. Temporal Shift

The share of information between head and thorax angular velocities may be delayed. For example, the thorax angular velocity until a time point *t* − τ may be used to predict the angular velocity of the head at time point *t*. In that case, the thorax angular velocity contains enough information to forecast the head angular velocity. We apply the same procedure described above, but with temporally shifted head and thorax angular velocities.

The forecasting of the head angular velocity along the z-axis will be based on the body angular velocity during a time interval Δ*t*:

(4) $$\begin{array}{r} {\hat{\omega }}_{z}^{\text{head}}\left(t-\tau \right)=g\left(\omega_{z}^{thorax}\left(t-\Delta t\right),\cdots \;,\omega_{z}^{thorax}\left(t\right)\right) \end{array}$$

Here, τ is the time between the last observation used for prediction and the time at which the angular velocity of the head is predicted. Similarly, the backcasting of the head angular velocity along the z-axis will be based on the body angular velocity during a time interval Δ*t*:

(5) $$\begin{array}{r} {\hat{\omega }}_{z}^{\text{head}}\left(t-\tau \right)=g\left(\omega_{z}^{\text{thorax}}\left(t\right),\cdots \;,\omega_{z}^{\text{thorax}}\left(t+\Delta t\right)\right) \end{array}$$

τ is thus the temporal shift between observation and prediction. For forecasting τ is negative. For backcasting τ is positive.

## 3. Results

Flying insects are thought to coordinate their thorax and head motion in order to maximize head stabilization. They segregate their flights into saccade and intersaccade. We investigated methods aimed at evaluating two different aspects of head-body coordination during insect flight: (1) Head saccade identification and (2) head angular velocity prediction based on the time structure of thorax movements. We applied and tested these methods to the learning flights of bumblebees, *B. terrestris*.

### 3.1. From Thorax Angular Velocities to Head Saccades

**Figure 3:**
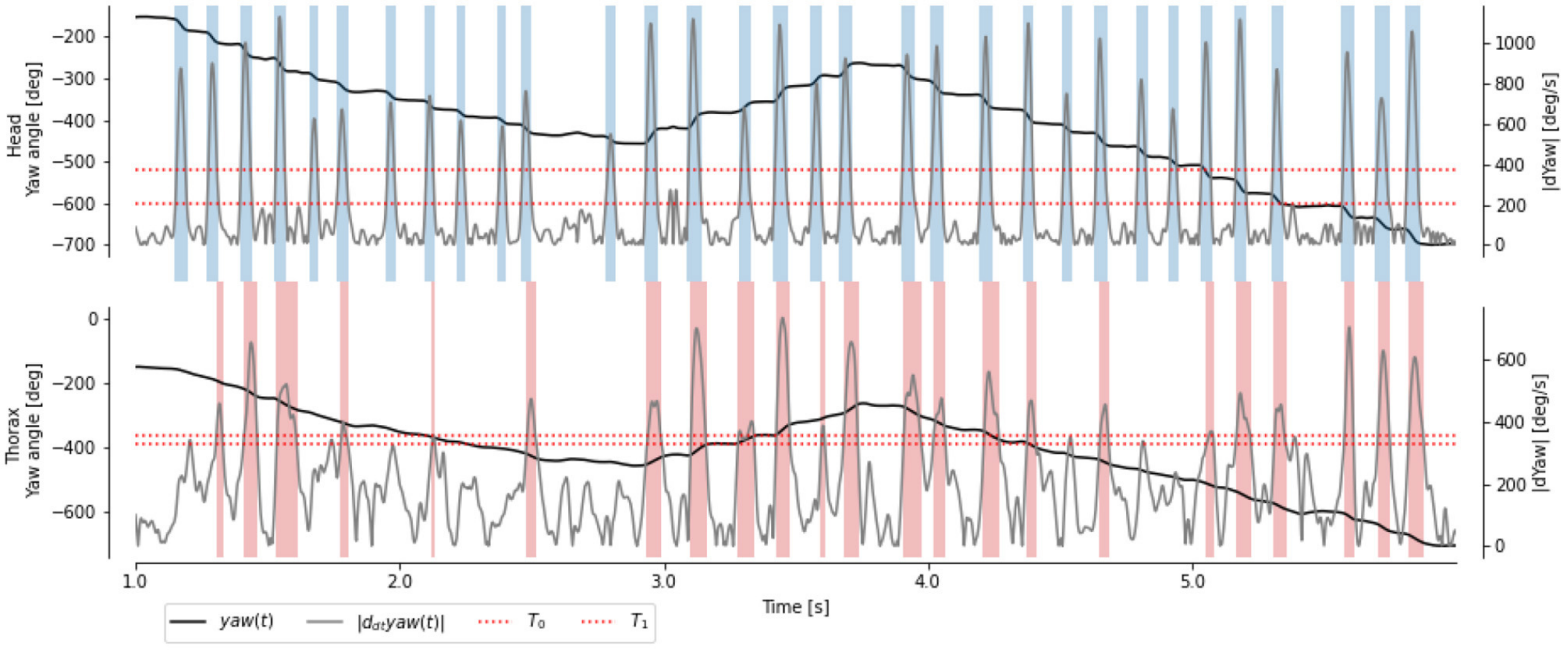
Time course of yaw angles and ω_*z*_(*t*) of the head and thorax of a learning flight. Head saccades (blue regions) are extracted with manually chosen thresholds (red dotted line). Body saccades (red regions) are extracted with thresholds to best match the head saccades.

In agreement with previous descriptions of flying insects' behavior, we observed that the angular velocity ω_*z*_ of the head is segmented into segments of high velocity (called saccade) and low velocity (called intersacade) Figure 3. The angular velocity of the thorax shows a similar pattern, but with a less neat segmentation between the intersaccades and saccades.

In the past, segmentation of insect flights into saccade and intersaccade was based on thresholding the angular velocity: angular velocities higher than the threshold are considered part of a saccade. Recording the head orientation of flying insects during free flights is technically demanding and has rarely been done (Van Hateren and Schilstra, 1999a; Riabinina et al., 2014). Thus, researchers have often only access to the orientation of the insects' thorax (Van Hateren and Schilstra, 1999a; Kern et al., 2012; Philippides et al., 2013; Ravi et al., 2013, 2019; Riabinina et al., 2014; Lobecke et al., 2018; Robert et al., 2018).

It thus raises the question: how well can we extract head saccades based on the thorax angular velocity? The first set of methods we investigated concerned the accurate classification of head saccades from thorax angular velocities.

#### 3.1.1. Decision Tree and Random Forest

Choosing a hard threshold to segment the time course of angular velocity based on the thorax is challenging, due to slower speeds during saccades and higher speeds during intersaccades. Thanks to the segmentation based on the head angular velocity, we can choose this threshold to maximize the accuracy (i.e., the percentage of frame correctly classified as saccade and intersaccade). Despite an optimally chosen threshold, we observed that many frames are incorrectly classified (compare misaligned red and blue stripes in Figure 3). The thresholding approach uses the velocity observed at time *t* to classify it as either saccade or intersaccade. However, head saccades have a time span of several 10 ms; thus, using neighboring observations may help classify the behavior.

Instead of choosing a single linear threshold, the field of machine learning offers algorithms that classify data. Here, the input is the angular velocity within a time window Δ*t* around a given time point *t*. The binary class to be predicted is either a head saccade or not a head saccade (intersaccade).

**Figure 4:**
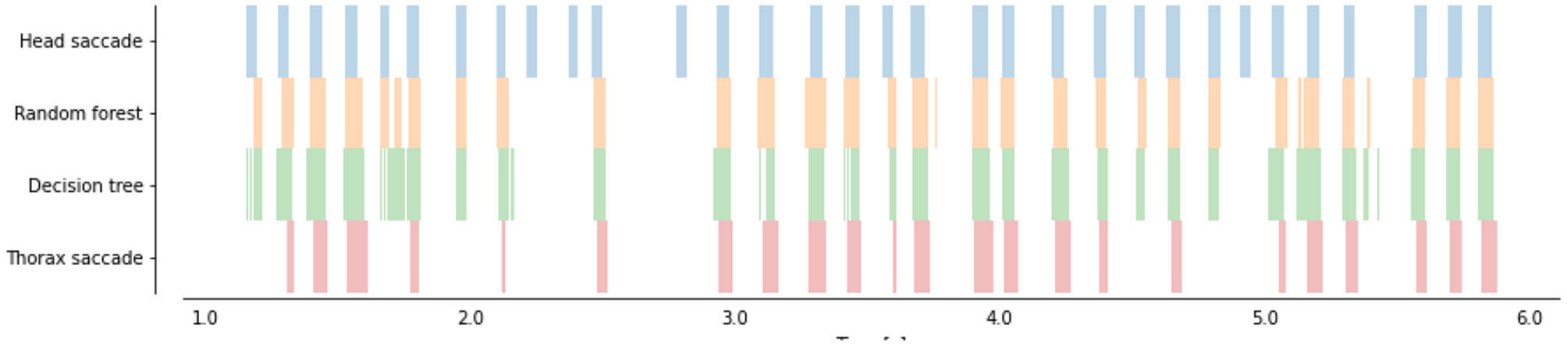
Time course of head saccades (blue) and predicted head saccades (orange and green) from thorax angular velocity ω_*y*_). Thorax saccades (in red) are extracted with a double thresholds (as in Figure 3). Two trained classifiers (a decision Tree, in green, and a Random Forest, in orange) were applied to the learning flight (which was not used for training) to predict head saccades.

Both classifiers DT and RD are classifying the thorax angular velocity with higher AUC than our benchmark (i.e., the thresholding method) Figure 4 on the test set. The Decision Tree, and Random Forest yielded an error rate of 11.91 and 11.26% (i.e., a 36.29 and 39.72% smaller error than the benchmark), respectively.

#### 3.1.2. Robustness at Low Sampling Rates

**Figure 5:**
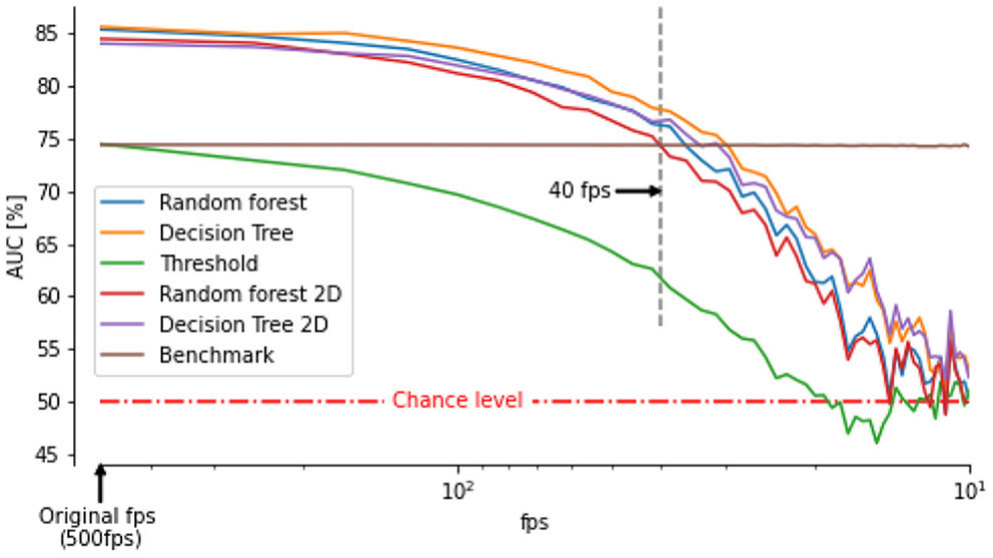
Area under the curve between saccade/intersaccade classifications from classifier or double thresholds on thorax angular velocity and head saccade/intersaccade classification as a function of frame rate. The benchmark is the classification from ω_*z*_ of the thorax based on the optimally chosen double threshold at 500 fps. The classifier outperform the benchmark for frame rates above 40 fps even when angular velocity of the thorax is determined by assuming zero pitch and roll orientation (classifier followed by 2D).

Our video footages were filmed at a relatively high frame-rate (500 fps) and captured using multiple perspectives to extract the 3D positions and orientations of the bee's head and thorax. However, recording at high frame rates and high spatial resolution requires special hardware not always affordable or available (for example during field experiments). Therefore, many experiments have been performed with frame-rate between 50 and 100 fps (Kern et al., 2012; Philippides et al., 2013; Ravi et al., 2013, 2019; Riabinina et al., 2014; Lobecke et al., 2018; Robert et al., 2018). At such a frame rate, data can usually be processed online (Straw et al., 2011; Stowers et al., 2017) or saved requiring reasonable space on hard drives. To assess the ability of our classifiers to identify saccades and intersaccades from low temporal resolution thorax orientation, we down-sampled our original recordings. We then interpolated the data with cubic splines to retrieve the 500 fps on which our classifiers were trained. The accuracy of the classifiers decreases with decreasing temporal resolution. Still, for frame rates higher than 40fps our classifiers perform better than (or as good as) our benchmark (Figure 5).

#### 3.1.3. Robustness to Single Camera Recordings

Obtaining the 3D orientation of the thorax requires the identification of at least three points on multiple views or, when sufficient visual features are visible, use advanced computer vision techniques for pose estimation from a single perspective (Graving et al., 2019). The orientation can, however, be approximated from a single perspective by making some assumptions. For example, assuming a null pitch and roll, the yaw orientation can be obtained from a single perspective view at the flying insect from above (Kern et al., 2012; Philippides et al., 2013; Lobecke et al., 2018; Robert et al., 2018; Ravi et al., 2019). We replicated this assumption on our data and assessed how well our classifiers could segment the flights into saccades and intersaccades. The classifiers still performed better than our benchmark for frame rates higher than 70 fps.

### 3.2. Predicting Head and Thorax Angular Velocity

Flying insects orchestrate the movements of their head and thorax in a timely manner, such that the head saccade and thorax saccade temporally overlap. The neurons controlling the head and thorax movements receive inputs from different brain areas (Schröter et al., 2007; Ibbotson et al., 2017; Steinbeck et al., 2020) One of these inputs could be an efference copy (i.e., a copy of an outflowing movement-producing signal generated by the motor system) of the head motion that affects the control of the thorax. The reciprocal would be an efference copy of the thorax motion affecting the control of the head. The efference copy signal needs to be processed and transmitted to another part of the bee's body, to affect the control of the targeted movement. If this were the case, we would expect two characteristics: (1) information is shared between the head and thorax angular velocity, and (2) the information at a given time *t* can be mapped to information at a later time *t* + τ.

#### 3.2.1. Predicting Head Velocity From Thorax Velocity (τ = 0)

**Figure 6:**
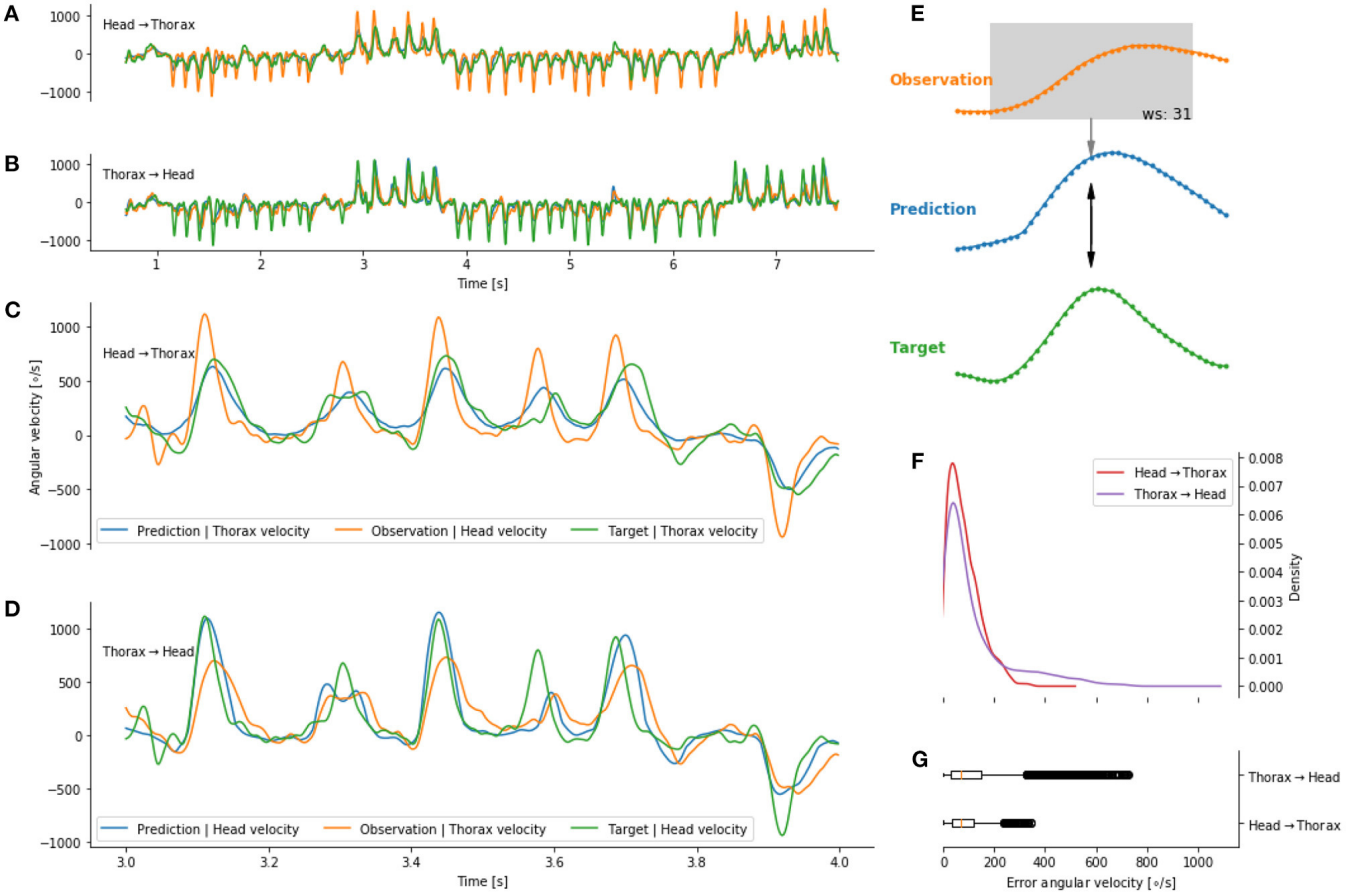
Predicting head and thorax angular velocity (without temporal shift) on a given flight not seen during training of the artificial neural network. **(A)** Prediction, in blue, of the thorax angular velocity from head angular velocity (orange). **(B)** Prediction, in blue, of the head angular velocity from thorax angular velocity (orange). **(C,D)** Zoom on **(A,B)**, respectively. The prediction (blue line) follow the target (green line). **(E)** Schematic of a prediction at a given time point. **(F,G)** distribution of error between predictions and targets.

We observed that the head and thorax angular velocity temporally overlap. It therefore seems likely that a mapping between the movements of the two body parts at τ = 0 exists. Dürr and Schilling (2018) used an artificial neural network (ANN) to investigate whether information from a given body part can be mapped to another. We used an ANN to map the head to the thorax angular velocity (and *vice versa*) without temporal delays (i.e., τ) Figure 6E. We observed that the predicted head angular velocity co-varies with the bees' head angular velocity (Figures 6B,D). A similar observation is made for the prediction of the thorax angular velocity from the head angular velocity (Figures 6A,C). The errors between the prediction and target are concentrated below 200 deg/s, i.e., below the variation of angular velocity during intersaccades. However, we observed that a prediction of the thorax angular velocity from the head angular velocity yielded lower errors than the reciprocal prediction (Figures 6F,G).

#### 3.2.2. Forecasting and Backcasting Head Velocity From Thorax Velocity (τ≠0)

**Figure 7:**
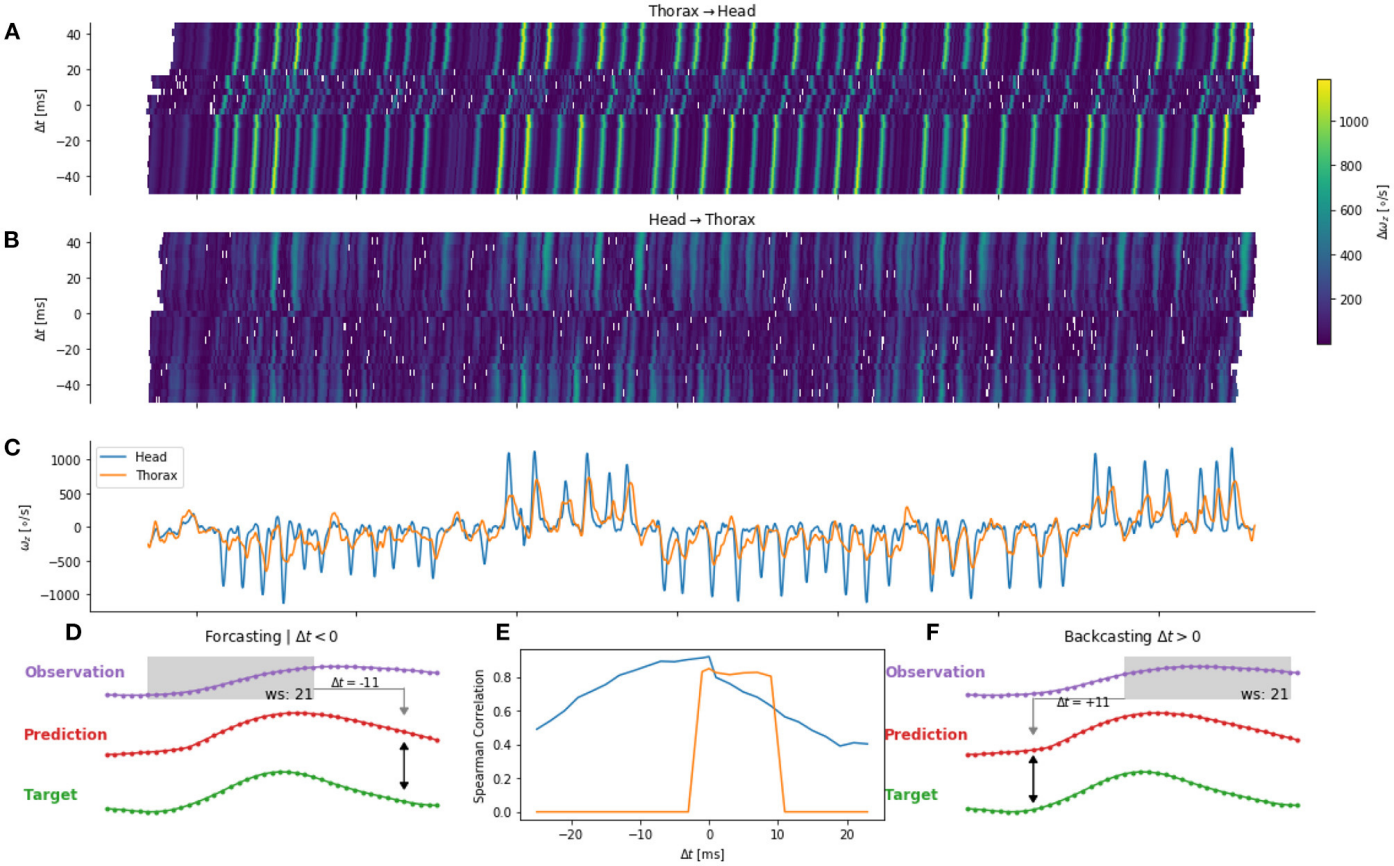
Error for one flight (validation set) for thorax to head **(A)** and head to thorax **(B)** prediction. Time course of the head and thorax angular velocity **(C)** are shown in blue and orange, respectively. **(D,F)** Example of a positive and negative shift of ±τ11 time steps of the observation. **(E)** Correlation between target and prediction for different temporal shifts.

We investigated the mapping of information between body parts for different delays τ. Similarly to the τ = 0 case, we used an ANN to map the angular velocity of one body part to another. However, the observation (for example, the head angular velocity) was temporally shifted relative to the target (for example, the thorax angular velocity). When τ is negative, the observation occurred before the target. Thus, this observation could be used to control the behavior of the target. For example, the thorax velocity at time *t* is sent as an efferent copy to the head control arriving at *t* − τ. We will refer to this case as *forecasting* (Figure 7D). If, τ is chosen to be positive, the observation occurred after the target, hence we will speak of *backcasting* (Figure 7F). This case mainly serves the purpose of avoiding over-interpretation of the results and will be later discussed.

We observed that the prediction error varied as a function of time (Figures 7A,B). By comparing the time course of the errors (Figures 7A,B) and the observation (Figure 7C), we observe higher errors during saccades. Such a pattern may be observed, when the network predicts a zero angular velocity, because during intersaccades the angular velocity is close to zero. This may happen when the potential relationship between the two body parts cannot be learned by the network. The prediction from the network and the target are, in this case, not correlated. Thus, we quantify the error as a function of the time-shift by using the correlation between the target (e.g., head angular velocity) and its prediction (e.g., head from thorax angular velocity).

The prediction of the head angular velocity correlates well with the network prediction, but the correlation is lower for large temporal time shifts. We observe a plateau between τ = −12ms and τ = 0ms (i.e., for forecasting), and a sharp decrease for τ > 0, and a smooth decrease for τ < −12*ms* (Figure 7E). In contrast, the prediction of the thorax angular velocity correlates poorly with the network prediction, except between τ = 0 and τ = +12 ms, i.e., for backcasting.

It, therefore, seems that an efference copy of the head angular velocity is sent to control the thorax angular velocity.

## 4. Discussion

To understand how different body parts work together and interact with each other, their kinematics must be recorded. However, some small animals can move very fast (for example a bee performing a saccade). Thus, the necessary equipment to track such fast movements, is often not available. Hence, the orientation of the thorax, which is relatively easy to track, is often used as a proxy for head orientation. However, this proxy is prone to errors. We used data of head and thorax orientation during learning flights of bumblebees and developed two methods to reduce this error. The coordination of head and thorax is of particular interest for understanding how information is gathered and processed by the bee, for example, the estimation of the distance to surrounding objects during intersaccades. Our first method predicts the saccades of the bee's head from the time course of thorax movement. Usually head saccades are identified by applying a threshold on the thorax angular velocity. This method does not lead to optimal results. Therefore, we trained a decision tree and a random forest classifier to automatically determine when head saccades take place, given the time course of thorax orientation. We were able to reduce the mis-classifications made when choosing the threshold manually from 39.72 to 11.26 %. A binary classification between saccades and intersaccades is likely not sufficient to fully understand the coordination between body parts in detail. Dürr and Schilling (2018) showed that it is possible to use an ANN to map the posture of one leg of a stick insect to the posture of another. We successfully used this concept and applied it to predict the orientation of a bee's head angular velocity from that of its thorax. Furthermore, we added a temporal component (forecasting or backcasting) to analyse how head and thorax work together. Our findings show that for a temporal shift of up to 10 ms it is possible to predict the head orientation from thorax orientation. If the shift is bigger the error increases drastically.

### 4.1. Technological Aspects

Many moving animals and robots alike actively shape their gaze to extract relevant information about their surroundings (Egelhaaf et al., 2012; Wisniewska et al., 2012; Tuhkanen et al., 2019). For example, during translation, the apparent motion of an object informs the agent about its relationship in space to the surroundings. If one can track the head of an animal during its course of movement, one could pinpoint for example when distance information is perceived. However, tracking the animal as a whole might already pose a challenge, rendering the tracking of specific body parts a nearly infeasible endeavor. For instance, researchers have to restrict the space of the recording area and use expensive recording devices to allow high spatio-temporal resolution. The spatial limitations constrain research potentially to only a part of the behavior. Additionally, researchers are often obliged to manually track the body parts of interest, a labor-intensive process which introduces a potential experimenter bias to the data. Our method solves this issue by allowing us to infer the timing of head saccade/intersaccade from thorax orientation at a rather low temporal resolution (40 fps) and a spatial resolution sufficient to record only the thorax. For example, in the case of bumblebee learning flights, by only tracking body orientation, one could predict head orientation and, thus, unravel when and how distance information might be learned about the nest-hole environment to enable later returns (Doussot et al., 2020a). Similarly, when bumblebees are crossing a difficult passage such as a gap in a wall (Baird et al., 2016; Ravi et al., 2019), head movements may be used to extract relevant information. By studying head movements, one can interpret the flight sequence performed at the entrance of this aperture. We should note, however, that we cannot infer the complete orientation (yaw, pitch, roll) of the head from thorax movements; therefore, our method cannot account to assess roll and pitch rotations in these behaviors.

### 4.2. Other Machine Learning Approaches

Our two methods are based on supervised machine learning techniques. First, for our classification task, we used two different classes of models. We decided to use an easily trainable Decision Tree. However, this class of models brings several disadvantages, of which one is its high variance, meaning small changes in data can lead to drastically different optimal trees. We therefore employed a more powerful class of models, Random Forests, which extend the concept of Decision Trees, mitigating the previously mentioned problem up to a certain amount. Unfortunately, Random Forests often become very complex and are not as clearly to be interpreted as Decision Trees. In any case, both models have shown that classification is possible, indicating two possibilities to pursue in future research.

First, one could utilize feature engineering. All our models operated directly on the input data, but research shows that feature engineering can improve performance (Wang et al., 2016; Banerjee et al., 2019). Second, more sophisticated (but potentially also data demanding) approaches could be employed. An example, is the use of Convolutional Neural Networks, which have been shown to work on time series classification with some minor adjustments (Gamboa, 2017).

Second, for our prediction task, we used a feed forward neural network inspired by the work of Dürr and Schilling (2018) to predict head orientation from body orientation. Alternative forecasting methods, such as ARIMA and Echo State Networks, have been used to forecast thorax position from previous observations (Meyer et al., 2018). These approaches may also be used to study the coordination of multiple body parts. Indeed, in the field of machine learning, the problem presented in this paper is coined time series classification, which spawned an extensive research endeavor (for a review see Bagnall et al., 2017).

### 4.3. Biological Implications

Our method to estimate the head angular velocity from the thorax angular velocity relied on an artificial neural network. The network can only relate the two angular velocities when a function between them exists and the network design can capture such function (Csáji et al., 2001). For example, by increasing the number of neurons in the network more complex functions can be found (Dürr and Schilling, 2018). The existence of such a function, embedded in the network, implies that input and output of the network share information. We applied this idea to the head and thorax angular velocities and found that indeed information is shared between the two.

Information in a dynamic system (such as the thorax and head control) is transmitted through the system either directly between subsystems or via another subsystem (for example a brain region). Hence, the sharing of information between subsystems in a dynamic system (e.g., between head and thorax) can be due to a common source (e.g., a central pattern generator; Guertin, 2013) or efference copies sent from one target to another (Straka et al., 2018). In both cases, a signal needs to be transmitted. Hence, generating a delay between the source and the destination will occur. By temporally shifting the recorded source (for example the head) relative to the target (for example the thorax) and using an artificial neural network, we studied the information flow. We found no evidence that the information flows from the thorax to the head.

A unidirectional flow of information between head and thorax can be observed when a source S (e.g., visual perception) is coupled to the head control (H), and also drive to some extent thorax control (T) in a way that the source can predict thorax but not vice versa (Granger, 1969; Diebold, 2006). Then due to transitivity of causality (if *H* → *S* and *S* → *T*, then *H* → *T*), information flows uni-directionally from H to T either directly (without a source) or indirectly (via the source) (Sugihara et al., 2012; Ye et al., 2015). Whereas, a direct flow implies biologically an efference copy, an indirect flow implies common brain regions implicated in the control of the two body parts. In addition, a source *S* (e.g., the brain) could control the head *H* and thorax *T* movement. Our method can not disambiguate between an efference copy or a feedforward control from or via a common brain region, but may be used to suggest how the information does not flow in the system.

Our data suggests no efference copy sent to the wing motor neurons to control head-yaw velocity during saccades. However, in the context of roll stabilization, to align the field of view to the horizon line (Raderschall et al., 2016), it has been suggested that head stabilization in flight is controlled by a feed-forward signal, where a copy of the command signals to the wing motorneurons is sent with an opposite sign to the head position control system (Viollet and Zeil, 2013) but also with the visual horizon (Goulard et al., 2015). Interestingly, these observations suggest different flows of information and pathways controlling the head and thorax' movements underlying roll stabilization and a saccadic gaze strategy.

The use of an artificial neural network to test the predictability of the output of one subsystem from the other informs not only about the potential causal relationship between these systems, but also provides a function relating the two. Thus, predictions can be made based on this function when information about one of the systems is not available.

## 5. Conclusions

We built our model on the bumblebee's complex maneuvres performed during learning flights. These flights are convoluted and are significantly different from the flights shown by bumblebees crossing a cluttered environment (Srinivasan, 2011), for example. The saccadic flight and gaze strategy at high temporal frequencies and high velocity is observed in multiple species. Flies or honeybees, for example, perform saccades similar to bumblebees. Flies' saccades tend to be slightly faster ( 5000°/*s*) than the ones of bumblebees (Van Hateren and Schilstra, 1999a; Braun et al., 2012). In contrast swimming seals turn their head with an angular velocity of only up to 100°/*s* in a saccadic manner (Geurten et al., 2017). Our saccade-intersaccade classifier based on the thorax angular velocity of bumblebees is likely to require retraining before being applied to other species. Nonetheless, because precise predictions could be made despite the complexity of the learning flights, it is likely that the presented methods can be adapted and extended to evaluate data from different animals.

Similarly to the classifier, our method to study the orchestration of movements of different body parts can be used to study movements of different animals, but the resulting trained networks are likely usable only for closely related datasets. It would, therefore, be of interest to apply this method to different species and behavioral assays to test its broader applicability.

## Data Availability Statement

The original contributions presented in the studz are included in the data publication (Doussot et al., 2020b).

## Author Contributions

LO and OB conceived, designed, and implemented the machine learning methods. CD and OB designed the bumblebee experiments. CD conducted the experiments, reviewed the trajectories, and extracted the time-course of the head and thorax orientations. SM and CD reviewed the implemented methods. All authors contributed to writing and reviewing manuscript, and approved the submitted version.

## Conflict of Interest

The authors declare that the research was conducted in the absence of any commercial or financial relationships that could be construed as a potential conflict of interest.
