# Supplementary material for: Analysing Head-Thorax Choreography During Free-Flights in Bumblebees
Source: Front Behav Neurosci. 2021 Jan 12;14:610029. doi: 10.3389/fnbeh.2020.610029 (PMC7835495; doi:10.3389/fnbeh.2020.610029)
Supplement: Supplementary file 1 [file Data_Sheet_1.PDF]

Analysing head-thorax choreography during free-flights of bumblebees, *bombus terrestris*.

1 SUPPLEMENTARY TABLES AND FIGURES

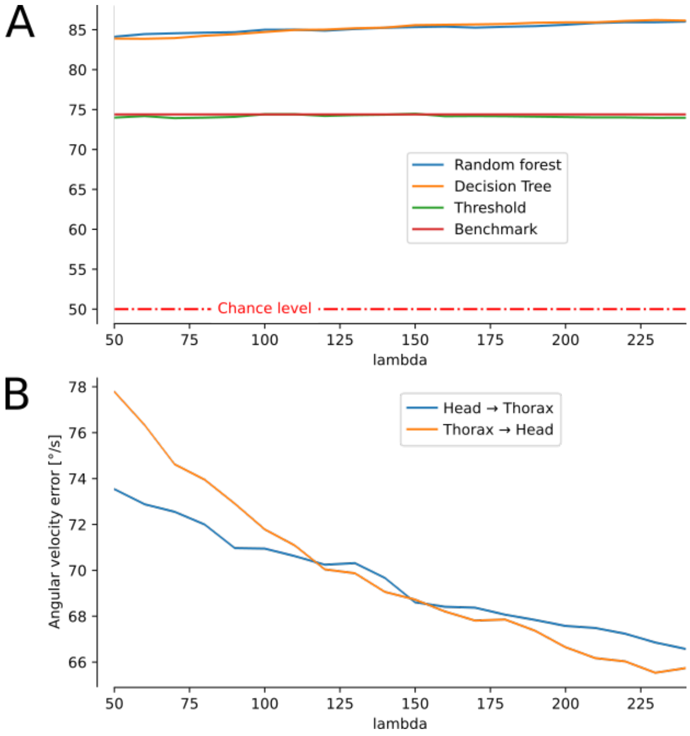

Figure S1. Effect of the smoothing parameter λ on the accuracy of the saccade-intersaccade classifier and and angular error of the neural network, respectively. The timecourse of the head and thorax orientation was filtered with a cubic spline with parameter λ before developing the two methods. A) To test for the robustness of the saccade-intersaccade classifier against the smoothing parameters λ, we evaluated the accuracy of the classifier (Thresholding, Decision tree, and Random forest) for λ varying between 50 and 250. We observe a slight increase with decreasing λ, and less variance for the Random forest classifier B) We tested the robustness of the neural network in predicting head's velocity from thorax's velocity (and vice versa) for different λ. We observe a decrease in angular error between network prediction and head angular velocity with increasing λ.

Table S1. Overview of the parameters used for the two methods, classifier and fore-backcasting.

	Parameter	Value/Range
Filtering	λ (smoothing parameter)	150
Classifier	Δt (window size)	$\in \{1, 3, 5, \cdots, 53\}$
	D (depth)	$\in [1, 20]$
Fore-backcasting	Δt (window size)	$\in \{1, 3, 5, \cdots, 53\}$
	N (number of neurons)	$\in \{1, 2, 4, 8, 16, 32, 64, 128\}$
	λ (regularization term)	0.5
	ϵ (avoid division by zero)	0.1
	τ (temporal-shift)	$\in \{-25, -23, \cdots, 23, 25\} \cup \{0\}$

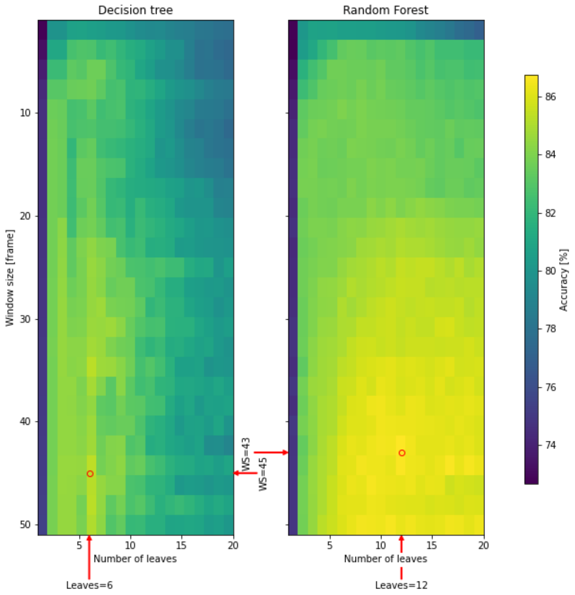

Figure S2. Accuracy of the Decision tree and Random forest on the validation datasets for different parameters. The windows size Δt was varied in $1, 3, 5, \cdot, 53$ (x-axis), and the depth of the trees D was varied from 1 to 20 (y-axis). Color represent the accuracy on the validation datasets. The red dot indicate the chosen parameter $Deltat$ and D for evaluating the model on the test set.
